# Translation of The Otago Exercise Program for Adoption and Implementation in the United States

**DOI:** 10.3389/fpubh.2014.00152

**Published:** 2015-04-27

**Authors:** Tiffany E. Shubert, Matthew Lee Smith, Marcia G. Ory, Cristine B. Clarke, Stephanie A. Bomberger, Ellen Roberts, Jan Busby-Whitehead

**Affiliations:** ^1^Center for Aging and Health, University of North Carolina, School of Medicine, Chapel Hill, NC, USA; ^2^College of Public Health, The University of Georgia, Athens, GA, USA; ^3^Department of Health Promotion and Community Health Sciences, Texas A&M School of Public Health, College Station, TX, USA; ^4^University of North Carolina Center for Health Promotion and Disease Prevention, Chapel Hill, NC, USA; ^5^Division of Geriatrics, University of North Carolina, School of Medicine, Chapel Hill, NC, USA

**Keywords:** fall prevention, health promotion, physical therapy, balance, aging, policy

## Abstract

**Background:**

The Otago Exercise Program (OEP) is an evidence-based fall prevention program developed, evaluated, and disseminated in New Zealand. The program was designed for delivery in the home by physical therapists (PTs). It was not known if American PTs would require additional training and resources to adopt the OEP. This article describes the process of translating the OEP for dissemination in the US. Processes included reviewing and piloting the New Zealand training materials to identify implementation challenges, updating training materials to be consistent with American physical therapy practices, piloting the updated training materials in an online format, and determining if the online format reached the target PT audience.

**Methods – Process Activities:**

The New Zealand manual was reviewed by expert American PTs and a training webinar was piloted with 56 American PTs. Feedback suggested that the program itself was understood by PTs, but training materials required modification related to documentation and reimbursement policies. Additional content was developed and integrated into an online training module. The online training was piloted and then deemed adequate by seven PT subject matter experts. The online training was launched in March 2013. Demographic and practice data were collected to characterize the PTs attending the online training as well as perceived barriers and facilitators to implementation (*n* = 522). Perceived facilitators include the effectiveness of the OEP to facilitate adoption, but the lack of agency support, billing and reimbursement challenges pose a significant barrier to OEP implementation.

**Conclusion:**

The OEP required additional information to facilitate adoption by American PTs. Online training that specifically targets PTs appears to effectively reach the target audience and be well received by participants. More research is required to determine the impact of online training on a PT’s adoption and implementation of this material into their practice.

## Introduction

Older adult falls are a significant public health problem (1). The reasons why older adults fall are complex and typically a result of multiple, interacting risk factors unique to the individual as they interact with their physical environment (2). The most common risk factors for falling are leg muscle weakness, difficulty walking, polypharmacy (too much or the wrong type of medications), cognitive impairment, vision impairment, and challenges within the environment (3). Of greatest concern are the falls experienced by those aged 75 and over. It is estimated that 50% of adults in this age group fall annually (4). These falls result in the greatest number of visits to healthcare providers and significant morbidity and mortality (5).

Given the extensive and complex nature of falls among older adults, interventions to prevent falls and related injuries have been studied for over two decades. Several fall prevention programs have been developed, tested, and proven effective to reduce falls among community-dwelling older adults (6). To facilitate the dissemination and implementation of these programs, the Centers for Disease Control and Prevention (CDC) published “Compendium of Effective Fall Interventions: What Works for Community-Dwelling Older Adults” in 2008 (7), with a second edition in 2010 (6). The second edition of the Compendium lists 22 interventions that have effectively reduced the rate of falls or fall-related injury. Each intervention includes a summary of the outcomes, program setting, target audience, content (key elements, frequency, and duration), and delivery system (who is qualified to deliver, level of training required). Of the 22 interventions, only three have incorporated and expanded the key elements into an implementation manual and training system to ensure program delivery with fidelity across community (Tai Chi: Moving For Better Balance and Stepping On) (8, 9) and home-based (the Otago Exercise Program – OEP) settings (10).

These three programs target older adults with the physical and mental abilities to live in non-institutional settings. Tai Chi is most appropriate for those older adults with the greatest mobility skills (11), Stepping On is for those older adults who are transitioning to be less mobile (12), and the OEP is the most effective for those older adults who are the least mobile and at the highest risk of falling (6). The OEP target audience may have limited mobility and access to group exercise settings, which differs from the other two programs in that it was designed to be delivered in the home (10).

The OEP was developed and evaluated in New Zealand in the late 1990s and proven effective in randomized controlled trials at reducing falls in high-risk older adults by 35% (13, 14). Due to the complex medical conditions inherent in the target audience, the OEP was delivered by healthcare professionals. The creators of the New Zealand OEP deemed that physical therapists (PTs), who receive extensive training in musculoskeletal rehabilitation, should at a minimum supervise, and ideally implement, the OEP (10). PTs have the training and expertise to evaluate an individual’s risk of falling; identify additional medical risk factors such as orthostatic hypotension, polypharmacy, arrhythmia; refer to other healthcare providers to manage risk; and prescribe and progress an older adult through a fall prevention program (15).

The OEP is an innovative model of low frequency of physical therapy sessions over a long duration. The original program was delivered in six visits over a year. The first four visits are in the first 2 months of the program (i.e., the initial visit, a visit a week later, then a visit 2 weeks later, then 4 weeks later); then follow-up visits are conducted at 6 and 12 months with monthly “check-in” phone calls between (13, 16). This type of model sets the stage for the patient engagement and ownership of their exercise program. The program only works if the patient does the exercises. The OEP achieves that goal with over 35% of participants stating they perform the exercises three times a week 1 year after the start of the program (13).

Given the robust results of the OEP, and the above average adherence and compliance rates, the CDC selected the OEP as one of three evidence-based fall prevention programs for dissemination in the United States. The implementation and dissemination materials for the OEP were developed in New Zealand. These materials offered a concise summary of the research supporting the OEP and step-by-step instructions about how the program was prescribed (10). However, the New Zealand manual did not account for policies and practices unique to the American healthcare system, nor did it provide any guidance about how to integrate the OEP into the workflow of a PT. It was not known if American PTs would require additional training and resources to adopt the OEP and implement it as intended.

The purpose of this article is to describe the process of translating the OEP for dissemination in the United States. Processes included reviewing and piloting the New Zealand OEP training materials with PTs to identify implementation challenges, updating the OEP training materials to be more consistent with American physical therapy practices, piloting the updated training materials in an online format, and then determining if the online format reached the target audience of PTs who work with frail older adults.

## Methods – Process Activities

### Translation of the Otago Exercise Program for dissemination in the United States

The OEP was developed and tested for dissemination and implementation in a country with a nationalized healthcare system. A manual to describe the implementation process was published in 2003 by the program developers (10). Before dissemination in the United States, it was deemed necessary to review all training materials and make modifications to support adoption and implementation by an American audience. Part of translation plan developed by the American team responsible for translating the OEP was to create and integrate a centralized system to offer education and training to PTs.

The following plan was deployed to review and revise the OEP manual and training materials for dissemination in the United States:

1. PTs with expertise in fall prevention and implementation of the OEP were to review the materials and identify any revisions necessary to support program adoptions
2. Pilot a real-time webinar based on the revised manual for American audiences amongst a small group of PTs from three states – Oregon, Colorado, and New York that were participating in the Centers for Disease Control Fall Prevention Pilot Project
3. Identify “lessons learned” from Otago implementation based on feedback from the webinars
4. Revise training materials based on lessons learned
5. Develop an online training program for broad dissemination in the United States
6. Pilot training with a small group of practicing PTs for feedback
7. Revise and deploy online training
8. Determine if online training was reaching the target audience of PTs most likely to adopt and implement the OEP in their practice settings.

### Revisions specific to American PTs

Expert PTs (T. Shea and T. Shubert) who had extensive knowledge of the OEP implementation both in the United States and in New Zealand worked with one of the OEP program developers (C. Robertson) to review the Otago Exercise Programme Manual (10). Revisions were made to the original manual. A United States version of the OEP Manual was released and made available in early 2012 (http://www.med.unc.edu/aging/cgec/exercise-program). The content of this manual was presented in a 1-hour training webinar offered four times in 2012 to 56 PTs. The 56 PTs who attended had been recruited by their respective State Division of Public Health Units (OR, CO, NY) to participate in a project to implement the OEP as part of the Fall Prevention Pilot Project. Attendance at the webinars was the first step in that process, and they were recruited via personal invitation from their state partners.

The training webinars were designed to pilot the material. Throughout the course of each webinar, therapists were encouraged to ask questions either by telephone or using the online chat function. We anticipated that many of the questions would be about how to actually prescribe the program; however, questions and discussions were more about implementation differences between New Zealand and America and how to address these differences. The following themes were identified as common challenges to implementation throughout the webinars:

1. The theory and implementation of evidence-based health promotion programs were not common knowledge for PTs.
2. In the original OEP research studies, subjects were at risk of falls but not actually seeing a PT for a diagnosed impairment. In order for Medicare to reimburse a PT for an episode of care, there needs to be a diagnosed impairment that requires “skilled and necessary” physical therapy (17).
3. Subjects in the original OEP research scored at risk of falls. This criterion was used for PTs to implement the OEP as part of the plan of care. Given that patients required skilled therapy, they were often weak and required a dose of physical therapy before starting the OEP. This dose of physical therapy was necessary to improve their strength and mobility so that they would be able to participate at the appropriate frequency and duration.
4. The OEP exercises were not unique to physical therapy, but the low frequency of PT visits and long duration of the OEP was deemed be an innovative practice model. Typical courses of physical therapy follow a model for 2–3 times a week for a period of 4–8 weeks. There was concern from therapists that the OEP model with its low frequency and long duration would be considered outside of the acceptable course of therapy. Being outside of normative values may result in a “red flag” to be audited by Medicare.
5. The OEP offered an opportunity to standardize practice around fall prevention. The literature demonstrates significant variations in clinical care around falls. Standardizing practice was appealing to some PTs and distasteful to others (18, 19).
6. The OEP was delivered in the home; however, the policies for billing and reimbursement for Physical Therapy under Medicare Part A (Home Health) make it virtually impossible to implement Otago over a year period.
7. A new model of PT delivery of care, which has emerged, allows for delivering physical therapy in the home but billing under Medicare Part B (outpatient). Though this model allows for greater opportunity to deliver the OEP over the year-long period, the paperwork burden on the PT was still sizeable.
8. Webinars and online training were deemed as an acceptable mode of training by PTs.

Given the feedback from the webinars, the content from the New Zealand manual was deemed appropriate for teaching therapists exercise program specifics. However, they believed that implementation in the United States would require additional information about how to integrate the program into the workflow, given documentation, billing, and reimbursement requirements. It was also identified that therapists would benefit from additional background about the theory behind evidence-based programs and the research behind the OEP.

The feedback from the PTs was then incorporated into the training manual. The PTs who attended the webinar agreed that the content of the OEP did not need to be presented in a face-to-face setting because much of the actual program was common to both PT practice and education. It was deemed that an online medium would be acceptable to disseminate the training to PTs in the United States.

### Development of online training program

**Figure 1:**
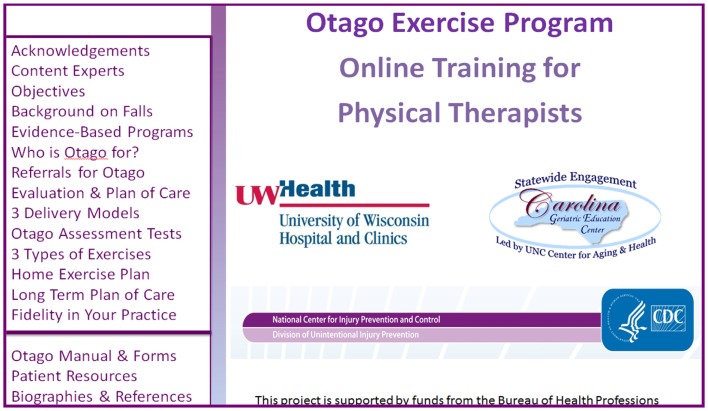
**Otago online training program and activities**.

The online curriculum was an adaption of the webinar and developed by the same authors. The curriculum incorporated “adult learning theory” using video, interactive assignments, and required posting to external discussion boards (Figure 1). The online version was developed into a power point and piloted by seven subject matter expert (SME) PTs – three knowledgeable about the OEP and four without prior experience of the OEP. All clinicians had at least 5 years of clinical experience.

The SMEs were invited to review the content from a select group of PTs who had received advanced certification in geriatrics, and who had contacted the researchers independent of the online training to learn more about the efforts to implement the OEP.

The SMEs were instructed to complete the course including outside assignments, quizzes, and a final exam. The SMEs then evaluated the following with open-ended questions (Table 1): (1) Course logistics – was it easy to find, navigate, complete? (2) Course content – was the information interesting, helpful, presented with fidelity to the original program? (3) Clinical usability/feasibility – could they apply this in their clinical setting? (4) Research – was it presented in a meaningful way? The responses were summarized and reviewed by course creators and independent external consultant.

**Table 1:** **Otago online pilot evaluation open-ended questions**.

Logistics
1. Describe how you found the course navigation to be. Was it easy to get around?
2. Were the directions clear to access phConnect?
3. How well did the quizzes cover the content in your opinion?
4. What was your opinion on the usefulness of the case studies presented in the videos?
5. How realistic did the case studies feel to you?
6. How easy was it to post questions on PH Connect?
Content
1. Tell me three things you remember from the content?
2. Was there anything that felt incompletely explained?
3. Was there anything that seemed too elementary?
4. ….or too advanced?
Your motivation
1. How different does the Otago Exercise Program feel from your customary PT practice?
2. How likely are you to use some of what you learned in the online course?
3. How motivated do you feel as a result of this experience to start using the Otago Exercise Program with your patients?
Research
1. How convincing did you find the research we presented?
2. Is the push toward using evidenced-based programs in PT more important to you now than it was at the beginning of the course?
Fidelity
1. How strictly do you think PTs have to stick to the Otago Exercise Program?
What unanswered questions do you still have regarding
1. The assessment tests
2. Billing for Otago
3. Choosing the exercises
4. On the Otago schedule and continuum?

The SMEs reported that the course was acceptable and engaging. The training was deemed adequate in length (2–3 h) and appropriately priced ($25). The curriculum was easy to navigate. The content was acceptable and clinically relevant. The exercise videos and case studies were well received; however, clinicians with several years of experience (>5) felt that the video cases were too contrived and not realistic.

Subject matter experts listed the following additional concerns:

1. “The OEP is appropriate for clinical use, but I have concerns about billing, reimbursement, and program fidelity” (four SMEs made this statement)
2. “The OEP may be challenging to implement and deliver for therapists who are not in-home Part B providers” (two SMEs).

### Deployment of online training program

The feedback from the SMEs was collated and revisions to the course were made. The course was deployed in March 2013. The course was advertised via the University of North Carolina at Chapel Hill’s School of Medicine website, national listserv for PTs, and word-of-mouth. Key partners such as the American Physical Therapy Association and CDC informed various groups interested in balance and fall prevention. The online training program continues to be advertised through monthly postings on national listservs for PTs, quarterly webinars for the National Falls Free Coalition, and at national and international meetings and conferences.

To minimize cost barriers, the course was priced at $25. Upon completion of the course, the registrants received 2 Continuing Education Units (CEUs). Many states require PTs to attend and report a minimum amount of continuing competence training annually to renew their license. These courses are often expensive. We felt offering low-cost CEUs would add an additional incentive to therapists interested in completing the training.

Participants enrolled in the course via the Area Health Education Center Connect website. The course was described as a 3-hour experience, which could be started and stopped at any time. After registration, participants completed a demographic form including key characteristics about their clinical practice (e.g., number of years in practice, percent of caseload over the age of 65), a pre-assessment of confidence in skills, and a baseline test about falls knowledge. The course had three other mini-quizzes embedded into the content throughout the course: (1) knowledge assessment of standardized protocols for functional tests; (2) an assessment of ability to evaluate functional tests and prescribe appropriate exercises from the OEP; and (3) an evaluation to assess the mastery of the concept of fidelity. Participants were not allowed to proceed to the next course section until they had demonstrated mastery of the content per the quiz score. Upon completion of the course, but before participants were awarded CEUs, they had to pass a final exam of 10 questions with a minimum score of 80% and complete a post-assessment about confidence in skills; an intention to implement survey that included items about perceived barriers and facilitators; and an evaluation of the course presentation and content. All participants received a follow-up survey via email 6 weeks after completing the course to assess level of program implementation. All participant data were collected with tools embedded in the training. Data were exported, de-identified, cleaned, and analyzed at 6 and 13 months post deployment.

## Results

It was unknown if the PTs who registered for the course would be the target audience for adoption. The goal was for PTs who worked primarily with older adults to complete the course. It was also unknown if the perceived facilitators and barriers by a larger audience would be consistent with the pilot results from the webinars. To ensure that the target audience was actually reached, frequencies for trainee demographics and perceived facilitators and barriers were calculated using data of the first 552 PTs enrolled in the course.

### Characteristics of online OEP trainees

The characteristics of all the OEP trainees who completed the training in the first 11 months of deployment are described in Table 2. During that time frame, 552 PTs, physical therapy assistants, and students enrolled in physical therapy programs enrolled in the course, and 398 completed the training. Table 3 describes the characteristics of the trainees practice settings. Of the 398, 30% were not in practice. These individuals were either students, researchers, or from other professions. The remaining 279 were predominately therapists with significant experience in geriatrics (211 had over 8 years of experience working with older adults) and worked primarily in geriatric settings (75% of sample stated more than 75% of their caseload was over the age of 65).

**Table 2:** **Demographics of therapists who completed the online program (*N* = 398)**.

	% Sample
Age
20–29	23
30–39	21
40–49	20
50–59	29
60+	7
Gender
Male	25
Female	75
Race
White	89
African-American	1
Asian	5
Native American	1
Other	4
Practice setting
Rural	30
Suburban/urban	66
Other	4
Patient care
Full time	45
Part time	25
Not in practice	30

**Table 3:** **Characteristics of therapist practice (*N* = 279)**.

	%
Years in practice
≤3	14
4–7	10
≥8	76
Years working with older adults
≤3	14
4–7	16
≥8	70
Average # visits/week
0–9	17
10–19	27
20–39	46
>40	90
% of caseload age 65 or older?
<25%	4
25–49%	6
50–74%	15
>75%	75
**Experience with evidence-based health promotion programs (EBHP)**	
Ever referred?
Yes	36
No	61
I do not know	3
Which program? (Select all that apply)
Matter of balance (*n* = 25)	9
Stepping on (*n* = 15)	5
Tai Chi (*n* = 75)	27
Other (*n* = 21)	8

### Perceived facilitators and barriers to program implementation (Table 4)

Trainees were instructed to “Please estimate the degree to which each of the following items will *facilitate* your ability to implement Otago,” and were given a list of 11 potential facilitators. Facilitators ranged from administrative support (i.e., would supervisors pay for copying of materials and help support documentation) to payor policies (i.e., what were the local Medicare polices toward longer duration of treatments with low frequencies) to compliance issues (i.e., would patients actually do the exercises on their own?). Trainees were also asked to “Please estimate the degree to which each of the following items will be a *barrier* to your ability to implement Otago,” and were given a list of 14 barriers. Barriers ranged from getting weights for patients to co-pays to paperwork issues. Table 5 lists the top three perceived facilitators and the top 3 perceived barriers.

**Table 4:** **List of facilitators and barriers**.

Facilitators	Barriers
I have active support from my Agency’s administration	My agency does not have reimbursement or billing policies in place
I have an internal “champion” or key leader who is supportive of Otago	Current Medicare reimbursement practices do not support delivery of the program
My agency has enough staff member, skills, resources to support the work and phone calls	Poor patient compliance
My agency is/will be able to modify reimbursement and billing practices to fit Otago guidelines	My agency is not set up keep patients on caseload over an extended period of time
The program is low cost and does not need substantial resources to continue	My agency does not have a system for follow-up phone calls
The research data helped convince my Agency of the value	It is difficult to get weights for patients
The research data helped convince referral partners (physicians, accountable care organizations) of value	Patients will not continue with a different Part B provider
The research data and program structure helped convince me of the value	Patients unable or do not want to pay co-pays
My patients like the program	Medicare C payors will not cover Otago
The program is supported by community and state-based fall coalitions	No way to transition patient from home health to Part B
I am able to bill as a Part B provider	Agency does not have enough trained staff members, skills, resources to support the work
Other facilitators (please specify)	Agency leadership does not support the work.
	Turnover among therapists implementing Otago
	Other barriers (please specify)

**Table 5:** **Top three facilitators and top three barriers**.

	Not at all	Somewhat	A lot
**Facilitators**
I have active support from my Agency’s administration	23	95	155
The program is low cost and does not need substantial resources to continue	18	119	131
The research data helped convince my Agency of the value	19	118	130
**Barriers**
My agency is not set up to keep patients on caseload over an extended period of time	66	127	65
Patients unable or do not want to pay co-pays	38	160	59
My agency does not have a system for follow-up phone calls	94	114	50

## Discussion

This study described the process of translating a research-based intervention developed in a country with nationalized healthcare for use in clinical practice within the United States. This article described the process of translating the OEP to facilitate adoption in the United States. Inherent in this process was identifying the barriers to adoption presented by implementing a program developed in a different healthcare system as well as identifying and implementing solutions to these barriers. In addition to translating the intervention materials, this process included the development of an efficient and effective system to disseminate training to PTs. A secondary purpose of this project was to determine if online training was an acceptable and feasible mechanism to reach our target audience of PTs.

The process of translating an intervention developed and tested in another county was innovative, and our experience indicates that it may be challenging to overcome barriers imposed by implementing programs under different healthcare systems. Two unanticipated challenges unique to the American healthcare system became apparent during the translation process: (1) reimbursement issues and (2) current policies regarding frequency and duration of physical therapy treatment.

Significant changes in Medicare Home Health Payment Policies were implemented during the time period of 2010–2013 (20, 21). When the OEP was first selected by the CDC to disseminate, it was assumed that PTs in the home health setting would be able to deliver Otago as intended and be reimbursed for their services. However, in October 2011, CMS released “The Final Rule” for implementation in 2012 (20, 21). The “Final Rule” significantly changed reimbursement for home health rehabilitation services with the goal of assuring equal access to services and reduce financial gaming. In essence, the final rule limited an episode of home health to no more than 60 days (it can be extended but with much paperwork) and reimbursed therapists at lower rates as more therapy was utilized. The 60-day limitation, in conjunction with an increase in acuity of home health patients and a 3–8% reduction in reimbursements depending on the patient’s acuity, effectively made it impossible for home health therapists to deliver Otago with fidelity.

Alternative models proposed by the American translation team leveraged PTs that treat patients in outpatient settings and have the ability to keep their patients on caseload for a longer period of time; however, this poses a significant challenge to the fidelity of the program. Innovative models that have therapists work with patients in the home, but bill as an outpatient have been investigated and demonstrate promise. However, this model for delivering therapy is relatively new and does not have widespread penetration.

Despite the popularity of evidence-based programs among public health professionals serving older adult populations (22), clinicians such as PTs are often not familiar with such evidence-based programs. The concept of fidelity, or delivering a program as intended, was not familiar to the majority of learners. More than 64% of those who took the training had never referred or incorporated an evidence-based health promotion program into their treatment plan. Many therapists felt that a standardized program was not flexible enough to meet the needs of their patients. The gaps identified through the work with the SMEs and the pilot testing with the PTs indicated that OEP content would be easy to convey to PTs, but the implementation of the program with fidelity would prove to be a challenge.

In recognition of these challenges, the online training was revised to include several case studies to demonstrate different implementation models including a home health to outpatient and an outpatient only case. Additionally, we believe the online model afforded several advantages over the traditional face-to-face model: (1) cost-effectiveness – participants were charged $25 to attend versus a face-to-face course, which is typically $100–200; (2) reach – in the first 9 months of deployment, we had participants from all 50 states take the training; (3) community – participants were invited to other opportunities to support their work; and (4) convenience – participants could start and stop the training whenever they liked.

In the first 9 months, the online training appears to be an effective mechanism to target PTs who work primarily with aging patients. The program itself was advertised through a word-of-mouth, website, and a few physical therapy-based listserv. The “early adopters” who completed the program were those who would be considered “senior” therapists (in practice 8 or more years) and spent the majority of their clinical practice time working with older adults. This supports that our target audience was reached. One concerning item was that only 13% of the sample were categorized as “new” therapists (3 years or less of clinical practice). The low number of new graduates may reflect the demographics within the greater practice setting and that the majority of PTs in geriatrics are older and more seasoned (23).

The perceived barriers and facilitators to program implementation provided significant insights about the challenges of the OEP adoption and implementation. At the end of the online training, therapists were asked to rate the extent an item was considered to be a facilitator or a barrier to implementation. The top facilitator was support from Agency administration. Therapists who implement the OEP without agency support are responsible for procuring weights, copying home exercise program handouts, and ensuring all paperwork is completed correctly and in a timely manner. One therapist estimated the personal cost of implementing the OEP at about $50/patient. Agencies that supported the OEP created systems to procure ankle weights for patients to use as part of the exercise program and ensured that all photocopying costs were absorbed by the agency as opposed to the therapist. Agency support is critical for program success, and more efforts should be made toward demonstrating the value of the OEP at the agency level.

Barriers included system-based challenges to maintaining a patient on caseload, concerns about costs to the patients in the form of co-pays, and the inability to perform follow-up phone calls. Interestingly, the therapists who completed the training did not perceive the billing and reimbursement challenges to be a barrier to program implementation. This may be because the therapists were being asked to rate these items immediately upon completing the online training and before actually implementing the program.

## Conclusion

The implementation of standardized fall prevention programs into physical therapy practice is not as simple or as straightforward as anticipated. PTs are well versed in the content of the OEP but were not familiar with the frequency, duration, and standardization of the program. In general, PTs appreciated the effectiveness of the program, but there are challenges inherent to reimbursement for providing the OEP with fidelity to appropriate patients. Online training appears to be an effective way to disseminate the OEP to PTs who work with older adults; however, we anticipate that additional support and resources will be necessary for PTs to implement the OEP with fidelity to impact the nature of falls.

## Conflict of Interest Statement

The authors declare that the research was conducted in the absence of any commercial or financial relationships that could be construed as a potential conflict of interest.

This paper is included in the Research Topic, “Evidence-Based Programming for Older Adults.” This Research Topic received partial funding from multiple government and private organizations/agencies; however, the views, findings, and conclusions in these articles are those of the authors and do not necessarily represent the official position of these organizations/agencies. All papers published in the Research Topic received peer review from members of the Frontiers in Public Health (Public Health Education and Promotion section) panel of Review Editors. Because this Research Topic represents work closely associated with a nationwide evidence-based movement in the US, many of the authors and/or Review Editors may have worked together previously in some fashion. Review Editors were purposively selected based on their expertise with evaluation and/or evidence-based programming for older adults. Review Editors were independent of named authors on any given article published in this volume.
